# Designated prescribing practitioners: a theory-based cross-sectional study of stakeholders’ views on implementation of a novel pharmacy regulator mandated preceptorship model

**DOI:** 10.1007/s11096-022-01467-8

**Published:** 2022-08-11

**Authors:** Tesnime Jebara, Trudi McIntosh, Fiona Stewart, Adam Osprey, Rachel Bruce, Scott Cunningham

**Affiliations:** 1 School of Pharmacy and Life Sciences, Robert Gordon University, Aberdeen, UK; 2 NHS Education for Scotland, Glasgow, UK; 3 Community Pharmacy Scotland, Edinburgh, UK; 4 NHS Greater Glasgow and Clyde, Glasgow, UK

**Keywords:** Drug prescriptions, Education and education, Models, Pharmacy, Preceptorship, Social theory

## Abstract

**Background:**

Scottish Government is increasing independent prescribers (IP) in community pharmacy (CP). A new preceptorship model using IPs as Designated Prescribing Practitioners (DPPs) has been introduced.

**Aim:**

To investigate stakeholder views of implementation of a novel regulator mandated IP course preceptorship model.

**Method:**

A theory-based online pre-piloted survey of stakeholders including e.g. directors of pharmacy, prescribing, education leads, policy & strategy leads and CPs. Questionnaire development used Consolidated Framework for Implementation Research (CFIR) and a DPP Competency Framework. Data were analysed descriptively and presented with mapping to CFIR constructs.

**Results:**

Of ninety-nine responses 82.5% (80/97) responded ‘yes’ to ‘..abilities in reporting concerns..’ and 53.1% (51/96) indicating ‘no’ to ‘..anticipated issues with clinical and diagnostic skills’. CFIR related facilitators included agreement that; there was tension for change with 84 (85%) indicating ‘….urgent need to implement role …’, that incentives are likely to help (6566%) and small pilots would help (8588%). Barriers were evident related to ‘unsure’ responses about sufficiency of; DPP capacity (39/97, 40.2%), time (48/96, 50%) and support and resources (4445%) to undertake the role. Concerns were expressed with 81 (83%) in agreement or unsure that leadership commitment may be lacking and 48 (48.9%) were ‘unsure’ about availability of good training for the DPP role.

**Conclusion:**

There was DPP role positivity but expressed barriers and facilitators at policy, organisational and individual practitioner levels needing further consideration. Further research is warranted on uptake and embedding of the role.

## Impact statements

- This theory-based work shows that there is enthusiasm for and confidence in the ability of community pharmacists to impact practice development by undertaking preceptorship roles as a Designated Prescribing Practitioner (DPP).
- There is a need to further consider the identified barriers and facilitators for implementation of the DPP role including funding continuity.
- Further work should also consider the impact of uptake and consolidation of the DPP role in community pharmacy and other sectors of practice.

## Introduction

A key development for health professionals has been the implementation of prescribing by non-medical health professionals, including pharmacists in the United Kingdom (UK), United States of America (USA), Canada and New Zealand [1–4]. There is growing international evidence supporting the implementation of pharmacist prescribing (PP). A 2016 Cochrane review reported that pharmacist and nurse prescribers were as effective as medical prescribers with many comparable clinical and humanistic outcomes [5]. Similarly, a 2018 review on views and experiences around PP highlighted many benefits to patients, pharmacists themselves, and the overall healthcare system [6]. The aims of implementing non-medical prescribing (NMP) are to improve patient care, patient safety and access to medicines and make better use of the skills of health professionals [7].

It has been advocated that the nature and quality clinical supervision is a vital part of the development of clinical skills [8]. Internationally, it is common for healthcare professions to have structured support frameworks for support of practice-based learning [9, 10] and in the UK there are developing supervision models for advanced pharmacy practice [11]. This need for consideration of structures and processes within the organisational environment has also been shown in other countries [12, 13] and has relevance in primary and secondary care.

Clinical supervision is a key element for qualification as an independent prescriber (IP) in the UK. Pharmacists must undertake a Masters level course delivered by a higher education institution and accredited to educational standards mandated by the UK regulator, the General Pharmaceutical Council (GPhC) [14]. Part of this mandate is that courses have a 90 h period of learning in practice (PLP) which aims to develop and assess competence to prescribe [2, 14]. Traditionally this period was supervised by a Designated Medical Practitioner (DMP).

Given the increasing workloads placed on DMPs, the GPhC have updated their course standards to allow qualified non-medical prescribers to take on the PLP supervisor role, now termed Designated Prescribing Practitioner (DPP) [15]. The importance of a supportive infrastructure of trained and quality-assured practice-based preceptors as part of an overall educational governance framework has been highlighted [16]. The need for high quality, well defined supervisory or preceptorship roles in pharmacy experiential learning has been recognised [17, 18] and there is a paucity of evidence around the standardisation of training programs [19, 20].

These issues have been addressed by the Royal Pharmaceutical Society (RPS), the professional membership body for UK pharmacists, which has developed the DPP Competency Framework [21]. The framework is novel in that it is intended to be used by all healthcare professionals who will supervise IP trainees (Table 1). Given the important role of DPPs, and efforts to standardize this through the competency framework, it is important to explore the views of DPPs on the implementation of the model.

**Table 1:** Structure of the Royal Pharmaceutical Society Designated Prescribing Practitioner Competency Framework [21]

Section 1: The Designated Prescribing Practitioner
1 Personal characteristics
2 Professional skills and knowledge
3 Teaching and training skills
Section 2: Delivering the role
4 Working in partnership
5 Prioritising patient care
6 Developing in the role
Section 3: Learning environment and governance
7 Learning environment
8 Governance

Additionally, there are ambitious plans to transform the 1250 community pharmacies in Scotland to make them a first point of contact [22]. In September 2020, the innovative NHS Pharmacy First Plus service was launched in Scotland which enables CP based IPs to prescribe for common clinical conditions out with the scope of standard contracted NHS schemes and would otherwise require referral to other healthcare professionals such as a general practitioner [23]. The IP assesses and may prescribe, within their scope of IP practice, for acute common clinical conditions which may include but not limited to urinary tract infections, respiratory infections, ear, nose and throat, dermatological presentations, allergies and eye infections.

However, implementation of IP focussed services in community pharmacy are falling short of government targets [24]. In view of this and the total number of pharmacies with scope for expansion of IP services, there is an need to increase the number of community pharmacy IPs. In May 2022, in Scotland it was announced that an additional 186 IP course places would be offered on top of the currently agreed 244 in Scotland [25] with a consequent a need to increase the number of DPPs for clinical supervision. While this study focuses on the UK context there is tremendous scope and opportunity for the model of preceptorship for non-medical prescriber training to have relevance to the many other countries considering or already implementing non-medical prescribing.

## Aim

The aim of this work was to carry out a theory-based investigation of stakeholders’ views of implementation of a novel pharmacy regulator mandated preceptorship model.

## Ethics approval

Ethical approval (S282) was granted by Robert Gordon University, School of Pharmacy and Life Sciences on 16 Nov 2020. As an educational development and evaluation project, the study was confirmed exempt from full NHS ethical review by West of Scotland Research Ethics Service on 19 Nov 2020.

## Method

### Study design

A quantitative online survey was employed since this was deemed suitable for gathering large amounts of data across a large geographical area to describe samples and populations using a set of questions [26].

### Settings and inclusion/exclusion criteria

The intention was to generate data from a wide range of key stakeholders involved in the planning and delivery of IP services and workforce development in practice. These stakeholders were best placed to contribute to research on implementation of the DPP role. The key stakeholders targeted included; health hoard directors of pharmacy, prescribing leads, CP leads and education and training leads, Community Pharmacy Scotland (CPS) organisation personnel (Chief Executive Officer (CEO), policy & strategy lead, prescribing lead) and community pharmacists including those that were IP qualified and provided Common Clinical Conditions Teach and Treat Training Hubs. Members of the research team were excluded.

### Sampling frame, recruitment and sample size

The sampling frame included all individuals meeting the inclusion criteria. The names and contact details of stakeholders were collated by members of the research team using their professional networks. An invitation email was sent to stakeholders within all Scottish 14 health boards and CP for consideration and dissemination through networks including: health Boards, CP organisations, the RPS and community pharmacists. Professional role groups targeted are listed in Table 2. An invitation to participate was also shared through social media (Twitter and Linkedin) to raise awareness and reach as many relevant key stakeholders as possible. Since this was a national survey with inclusion of a broad range of stakeholders without a defined sample list the sample size and response rate were indeterminate. However, Scottish data for 2021 from NHS Education for Scotland (NES) indicate there were 4956 GPhC registered pharmacists and 1373 IPs (Personal Communication). Assuming 85% work in community pharmacy or primary care [27] the stakeholder target for IPs would be around 1200. Including other stakeholders with interest and experience of prescribing policy and education (say another 200) gives an estimated population sample of 1400. Using an online survey sample size calculator with: 95% confidence Level, 1400 population and 9% margin of error the ideal sample size is 110 [28].

**Table 2:** Demographic data of questionnaire respondents (N = 99)*

Demographic category	Number of respondents (%)
*Age*	
< 25 years	1 (1)
25–29 years	11 (11)
30–34 years	18 (18)
35–39 years	15 (15)
40–44 years	19 (19)
45–49 years	8 (8.1)
50 years and over	27 (27)
*Gender*	
Male	26 (27)
Female	71 (72)
Other	1 (1)
*Professional role*	
Community pharmacists	39 (40)
Prescribing leads	7 (7)
Directors of Pharmacy	4 (4)
Education and training leads	4 (4)
Primary care community pharmacy leads	2 (2)
Designated Medical Practitioners (DMP)	0
Others	41 (42)
*Stage of career*	
< 2 years qualified	2 (2)
2–10 years qualified	21 (21)
Over 10 years qualified	76 (77)
*Previous involvement in developing or delivering IP courses*	
Yes	18 (18)
No	80 (82)
*What is your status as a prescriber?*	
In training	5 (5)
Registered prescriber but never prescribed	1 (1)
Registered prescriber but not currently prescribing	13 (13)
Registered prescriber and currently prescribing	57 (59)
Planning to undertake training in future	11 (11)
No plans to undertake training	8 (8)
Not applicable	0
Other	2 (2)
*How long have you been as a prescriber?*	
less than a year	7 (7)
1–5 years	22 (23)
6–10 years	15 (16)
11–15 years	20 (21)
16–20 years	5 (5)
more than 20 years	3 (3)
Not applicable	25 (26)

^*^Some missing data, % response calculated on basis of number responding to each item

### Development of data collection tool

The data collection tool was informed by and developed from: the DPP Framework [21], Consolidated Framework for Implementation Research (CFIR) [29] and Organisational Change Manager (OCM) tool [30]. Each was used to support the underpinning of the questionnaire items. CFIR has 5 broad constructs [31]; innovation characteristics, outer setting, inner setting, characteristics of individuals and process. The OCM (which is based on CFIR) tool has sections titled: project launch, problem exploration, solution development and implementation and testing. The questionnaire tool included separate sections covering these. Part A covered awareness and views of the role focussed on innovation characteristics and characteristics of individuals. Part B covered experiences and views of implementation of DPP predominantly focused on the outer, inner setting and process constructs to explore barriers and facilitators to implementation process. A final section included demographic questions to help contextualise the responses. A mixture of response scales were used including yes/no/unsure, 5 point Likert Scales (see Tables 3, 4 and 5). The questionnaire was reviewed for face and content validity by members of the research team prior to piloting with 10 stakeholders with pilot responses were not included in the final dataset.

**Table 3:** Stakeholders’ awareness and views of the role based on the RPS DPP Competency Framework (*N* = 99)*

Statement	Number of responses (%)		
	Yes	No	Unsure
*Section 1 – The Designated Prescribing Practitioner*			
Do you anticipate any issues with potential DPPs meeting the competencies that state: *DPPs should be experienced and active prescribers in a patient facing role*?	27 (28)	58 (60)	11 (12)
Do you anticipate any issues with potential DPPs relating to the *competencies around clinical and diagnostic skills*?	29 (30)	51 (53)	16 (17)
The framework states that DPPs should have *competence in the scope of practice relevant to the IP trainee* – do you see any issues with this?	33 (34)	40 (42)	23 (24)
*Section 2 – Delivering the role*			
Do you feel there will be any issues in a community pharmacy context relating to the DPP competencies around partnership working and taking a multidisciplinary team approach?	35 (36)	40 (41)	22 (23)
Do you feel that DPPs supporting independent prescribing trainees in community pharmacy will be able to access other practitioners better placed to support some aspects of trainee’s learning?	50 (52)	6 (6.3)	40 (42)
Will DPPs for independent prescribing trainees in community pharmacy be in a position to identify and respond appropriately to concerns regarding the trainee’s practice or behaviour?	67 (69)	7 (7)	23 (24)
Do you feel that there will be sufficient DPP capacity to effectively deliver the role and support the aspiration for increasing numbers of independent prescribing trainees in community pharmacy to meet the demands for new services such as Pharmacy First Plus?	19 (20)	39 (40)	39 (40)
*Section 3—Learning environment and governance*			
Do you feel that DPPs for independent prescribing trainees in community pharmacy will be able to negotiate sufficient time to undertake the role effectively?	16 (17)	32 (33)	48 (50)
Will the DPPs for independent prescribing trainees in community pharmacy be able to create an environment that promotes equality, inclusivity, and diversity?	75 (77)	2 (2)	20 (21)
Will the DPPs for community pharmacy independent prescribing trainees in community pharmacy be able to understand their role in relation to the wider healthcare governance structures?	70 (72)	2 (2)	25 (26)
Do you feel that DPPs will be comfortable reporting any concerns about trainees through agreed processes between them and the university?	80 (83)	3 (3)	14 (14)
Do you feel that DPPs for independent prescribing trainees in community pharmacy will be able to negotiate appropriate support and resources to undertake the role effectively?	42 (43)	11 (11)	44 (45)

^*^Some missing data, % response calculated on basis of number responding to each item

**Table 4:** Confidence and competence in current community pharmacist independent prescribers’ abilities to take on the DPP role (*N* = 99)*

Ability	Number of responses (%)									
	Confidence					Competence				
	Fully confident	Somewhat confident	Neutral	Somewhat lacking	Not at all confident	Fully Competent	Somewhat competent	Neutral	Somewhat lacking	Not at all competent
Train, teach and/or supervise in practice	15 (16)	49 (52)	12 (13)	19 (20)	0	19 (22)	46 (52)	16 (18)	7 (8)	0
Apply different teaching methods	9 (10)	29 (31)	31 (33)	20 (21)	5 (5)	9 (10)	30 (35)	33 (38)	12 (14)	3 (3)
Articulate decision making processes and justify rationales for decisions	30 (32)	45 (47)	15 (16)	5 (5)	0	31 (35)	44 (50)	11 (13)	2 (2)	0
Use a range of methods of assessment	14 (15)	40 (42)	19 (20)	18 (19)	4 (4)	15 (17)	35 (40)	18 (21)	16 (18)	4 (5)
Facilitate learning by encouraging critical thinking and reflection	20 (21)	46 (48)	18 (19)	9 (10)	2 (2)	21 (24)	43 (49)	17 (20)	5 (6)	1 (1)

^*^Some missing data, % response calculated on basis of number responding to each item

**Table 5:** Stakeholders’ experiences and views of implementation of DPP (*N* = 99)*

Statement **	Number of responses (%)			CFIR Constructs
	Agree/Strongly agree	Unsure	Disagree/Strongly disagree	
*Phase 1—Project Launch*				
Leaders at the different Health Boards lack commitment to spend their time & resources to remove obstacles when they arise	47 (48)	34 (35)	17 (17)	Inner Setting > Readiness for Implementation Leadership > Engagement
Not moving to implementation of DPP in community pharmacy in Scotland is unacceptable	72 (74)	15 (15)	11 (11)	Inner Setting > Readiness for Implementation Leadership > Engagement
The aim of DPP implementation in community pharmacy in Scotland is unclear	34 (34)	20 (20)	45 (46)	Process > Planning
Policy makers at the Scottish Government lack commitment to making DPP implementation successful	25 (25)	55 (56)	19 (19)	Process > Engaging > Champions
Scottish pharmacy stakeholder groups have substantial power to make things happen in relation to DPP implementation	64 (65)	29 (29)	6 (6)	Process > Engaging > Champions
*Phase 2—Problem Exploration*				
National strategies have been developed to inform and involve community pharmacy opinion leaders	32 (33)	46 (47)	19 (20)	Process > Engaging > Opinion Leaders
Patients/public should be involved to understand the issues around having more DPPs to help expand prescribing in community pharmacy services	47 (48)	28 (28)	24 (24)	Process > Engaging > Key stakeholders: Patients/ Customer
Community pharmacy managers are committed to spend their time & resources to remove obstacles when they arise	31 (33)	36 (38)	28 (30)	Inner Setting > Readiness for Implementation > Available Resources
Changes in community pharmacy services mean there is an urgent need to implement the DPP role within Scotland	84 (85)	11 (11)	4 (4)	Inner Setting > Implementation Climate > Tension for Change
DPP implementation has been influenced strongly by our proven ability to adapt ideas from other settings to fit community pharmacy organisations way of doing things	49 (51)	35 (36)	13 (13)	Inner Setting > Culture
DPP implementation has been influenced strongly by pressures from outside community pharmacy organisations	38 (39)	42 (43)	18 (18)	Outer Setting > External Policies & Incentives
*Phase 3—Solution Development*				
DPP implementation does not conform to the opinions of respected community pharmacy experts	11 (11)	39 (40)	47 (48)	Intervention Characteristics > Evidence Strength & Quality
DPP implementation appears to have many more advantages than disadvantages	74 (76)	15 (15)	9 (9)	Intervention Characteristics > Relative Advantage
NHS service implementation approaches can be adapted to fit DPP implementation in community pharmacy	59 (6)	32 (33)	6 (6)	Intervention Characteristics > Adaptability
NHS service implementation approaches can be adapted to suit local needs and still retain effectiveness for DPP implementation in community pharmacy	60 (61)	34 (35)	4 (4)	Intervention Characteristics > Adaptability
Enough money is available to support identifying, developing and implementing solutions to facilitate DPP introduction in community pharmacy	9 (9)	49 (50)	40 (41)	Inner Setting > Readiness for Implementation > Available Resources
Enough money is available to support training potential DPPs	12 (12)	51 (53)	34 (35)	Inner Setting > Readiness for Implementation > Available Resources
*Phase 4—Implementation and Testing*				
The plan for implementing DPPs should be simple; having no unnecessary or overly complex steps	87 (89)	8 (8)	3 (3)	Process > Planning
The plan for implementing DPPs should have a clear and realistic time schedule	97 (99)	1 (1)	0	Process > Planning
IPs taking on the DPP role are unlikely to be supported by good training	18 (18)	30 (31)	50 (51)	Inner Setting > Readiness for Implementation > Access to Knowledge & Information
IPs taking on the DPP role should believe at the outset that they are confident and competent to do it well	87 (89)	5 (5)	6 (6)	Characteristics of Individuals > Self-Efficacy
Stakeholder incentives are likely to make the DPP implementation successful	65 (66)	30 (31)	3 (3)	Inner Setting > Implementation Climate > Organizational Incentives & Rewards
Clearly defined leadership roles are likely to make the DPP implementation successful	89 (91)	8 (8)	1 (1)	Inner Setting > Readiness for Implementation > Leadership Engagement
Clearly defined organisation structure and documented procedures are likely to make the DPP implementation successful	91 (93)	4 (4)	3 (3)	Inner Setting > Readiness for Implementation > Access to Knowledge & Information
Small pilots of DPP implementation should be set up to collect honest reactions from all stakeholders	85 (88)	10 (10)	2 (2)	Process > Reflecting & Evaluating

^*^Some missing data, % response calculated on basis of number responding to each item. ** The sections are categorised into four main phases as per CFIR based Organisational Change Manager (OCM) tool [20]. These include; project launch (planning, leadership and champions), problem exploration (stakeholder (opinion leaders/patients) engagement, tension for change, culture, resource availability, external policy influence), solution development (evidence strength, relative advantage, adaptability and resourcing) and implementation and testing (access to information/training, incentives and reflecting and evaluating)

### Data collection and analysis

Post validation and piloting, the final questionnaire was hosted on JISC Online Surveys. An online link to the questionnaire and the participant information sheet detailing the study aims and potential benefits of participation, confidentiality, etc. were emailed to a comprehensive list of key contacts within each of the identified stakeholder groups as outlined above.

To maximise response rate and reduce potential social desirability and other biases, the research team employed the strategies detailed by Edwards et al. [32]. The questionnaire remained open from 1^st^ March till end of May 2021. Data were exported to SPSS Statistics for Windows, version 28.0 (SPSS Inc., Chicago, Ill., USA) where is was checked, screened, cleaned and then analysed descriptively. Data were also mapped to CFIR constructs.

## Results

Ninety-nine responses were received from across the different Scottish health boards. Respondents’ demographics are presented in Table 2. The majority were female, working as community pharmacists, over 10 years qualified, and with no previous direct involvement in IP course development or delivery. However, the majority were qualified prescribers (82, 85%) and currently actively prescribing (57 (59%), with 43 (44%) having greater than 6 years prescribing experience.

### Stakeholders’ awareness and views of the DPP role based on the RPS DPP competency framework

The first section focused on stakeholders’ views and awareness of the DPP role and was based on the RPS DPP competency framework (Table 3). Overall, the majority of stakeholders were positive about potential DPPs’ abilities to report (*n* = 80, 83%) and respond (67, 69%) to any concerns about trainees. They did not anticipate any issues with the clinical and diagnostic skills of potential DPPs (*n* = 51, 53%) or their ability to work collaboratively within a multidisciplinary team (40, 41%). However, a high proportion of respondents identified potential barriers to implementation indicating that they were ‘unsure’ about sufficiency of; DPP capacity (39, 40%), time to undertake the role (48, 50%), support and resources (44, 45%) to undertake the role effectively. There was dichotomy of response to the issue of whether DPPs would be ‘…able to access other practitioners better placed to support some aspects of trainee’s learning’ with 50 (52%) responding ‘yes’ and 40 (41%) responding ‘no’ which represent a further potential barrier.

### Confidence and competence in current community pharmacist independent prescribers’ abilities to take on the DPP role

The majority also viewed positively potential DPPs’ ***confidence*** and ***competence*** in training, supervising, and assessing IP trainees (Table 4). Over half believed that potential DPPs are somewhat or fully ***confident*** in their ability to train, teach and/or supervise in practice (64, 67%), articulate decision-making processes (75, 79%), use a range of methods of assessment (54, 57%), and encourage critical thinking and reflections (66, 70%). In terms of ***competence*** to perform the role, the majority of respondents believed that potential DPPs were somewhat or fully competent to train, teach and/or supervise in practice (65, 74%), articulate decision-making processes (75, 85%), use a range of methods of assessment (50, 57%), and encourage critical thinking and reflections (64, 74%).

### Stakeholders’ experiences and views of implementation of DPP

The next section comprised a series of attitudinal statements to explore CFIR related potential barriers and facilitators to implementation of DPP for community pharmacists in Scotland (Table 5).

A facilitator linked to *‘project launch’* was that there were generally high levels of agreement around ability to champion DPP implementation with 64 (65%) in agreement that ‘Scottish pharmacy stakeholder groups have substantial power to make things happen …’. However, this was tempered by potential barriers linked to some concerns at local and national levels around leadership with 81 (83%) in agreement or unsure about ‘Leaders at the different Health Boards lack commitment …’ and 80 (81%) in agreement or unsure about ‘Policy makers at the Scottish Government lack commitment …’.

Facilitator were evident liked to *‘problem exploration’* and *‘solution development’* sections with a very high levels of agreement that there is tension for change with 84 (85%) in agreement that ‘…. there is an urgent need to implement the DPP role …’ and advantages and adaptability around development of approaches to DPP implementation with 74 (76%) in agreement that ‘DPP implementation appears to have many more advantages than disadvantages’. However, at this stage of the development process there were still concerns around barriers focussed on resource availability with 89 (91%) indicating ‘unsure’ or in disagreement that ‘Enough money is available to support identifying, developing, and implementing solutions …’.

CFIR related facilitators were also prevalent in the last section related to *‘implementation and testing’* almost all (97, 99%) in agreement that ‘… implementing DPPs should have a clear and realistic time schedule’ and the majority, 65 (66%) in agreement that ‘Stakeholder incentives are likely to make the DPP implementation successful.’ Further facilitation of implementation was felt to arise from leadership and piloting with 89 (91%) in agreement that ‘Clearly defined leadership roles are likely to make the DPP implementation successful.’ and 85 (88%) in agreement that ‘Small pilots of DPP implementation should be set up to collect honest reactions from all stakeholders’. However a final potential barrier was identified from the response to ‘IPs taking on the DPP role are unlikely to be supported by good training’ with 48 (48.9%) in agreement with or unsure about this item.

## Discussion

### Statement of key findings

In relation to stakeholders’ awareness and views of the role based on the RPS DPP competency framework, most stakeholders were comfortable with potential DPPs managing any concerns about trainees and did not anticipate any issues with clinical and diagnostic or collaborative working. Key facilitators in the implementation process included; there was strong support and a belief that there exists a tension for change with advantages outweighing any disadvantages. There was an indication that clearly defined leadership roles, conducting small pilots and offering stakeholders incentives would also help. Barriers to implementation were identified as; lack of sufficient DPP capacity, lack of ability to effectively negotiate for resources (e.g., access other practitioners, time, support) to undertake the role effectively. There were some concerns at local and national levels around leadership and the availability of resources including training support for those undertaking the IP role.

### Strengths and weaknesses

A strength is that only a limited number of countries have introduced NMP with none allowing non-medical professionals to take on the role of the work-based supervisor. Thus, this research is original and supports implementation of the DPP role. To promote robustness this work employed the RPS and a theoretical framework to ensure comprehensive coverage of different aspects related to development and implementation.

In terms of limitations, the response rate could be considered poor but is not unusual for such online surveys. The fact that dissemination involved use of key contacts and social media and was in the immediate post-pandemic period may have affected the response rate and introduced sampling bias. However, data from 99 respondents is close to the calculated ideal sample with a 9% margin of error [28] and therefore provides valuable insights to the topic area with some robustness and confidence in responses. We acknowledge potential bias from respondents having no previous involvement in developing or delivering IP courses but nearly three-quarters were qualified as prescribers and so had ‘experience’ of IP training through course completion. No DMPs completed the questionnaire thus their views and perceptions were not included and some participants may have started the questionnaire but failed to submit it. Lastly, data were collected from Scotland and might not be generalisable to other countries.

### Interpretation of findings

In other countries where models of NMP are being implemented there are no examples of a role such as the DPP, mandated by a pharmacy regulator and defined by a professional body.

### Characteristics of the DPP role

Forsyth and Rushworth have highlighted the need for consideration of standardisation of preceptorship models [33]. The DPP preceptorship model is well defined within a robustly developed multi-professional evidence based framework [15, 21]. In view of this, the CFIR construct of ‘Intervention characteristics’ is well covered and there is clarity about the expected ‘characteristics of individuals’ [29]. In this context, the majority of respondents indicated that they felt that potential DPPs would be confident and competent in the role.

In the context of NMP, concerns have been expressed about the competence of pharmacists in relation to clinical and diagnostic skills [3, 6, 20]. However, respondents in this study did not anticipate issues. This may be a consequence of increased coverage at undergraduate level in the UK [34] and internationally [35]. There has also been an increase availability in such training at post-graduate level through both work- based learning and simulation [36–38]. Additionally, in Scotland, availability of funding for NMP training is contingent on pharmacists also completing courses in consultation and clinical assessment [39].

### Facilitators for implementation of the DPP role

Facilitators predominantly focussed on aspects of the ‘inner setting’ and ‘process’ CFIR constructs [29]. Respondents were clear that the readiness for and extent of implementation success of the DPP role would be dependent on significant leadership engagement to ensure creating urgency, building a guiding coalition, and creating and communicating a vision [40, 41]

It has been shown that motivation to take on preceptorship in CP relates to making professional contributions and job satisfaction [42, 43]. Respondents considered that there existed a positive culture of adaptability and willingness for change within pharmacy in Scotland. A further process facilitator related was the recognised need to build in systems to gather information through testing, piloting and ongoing monitoring of implementation [44].

### Barriers to implementation of the DPP role

CFIR ‘inner setting’ and ‘external policy and incentives’ barriers focussed on aspects around availability of resources and the influence of this on readiness for implementation. Stakeholders identified human resource capacity issues to fully deliver on operationalisation of the DPP role. In 2022, additional course places have been funded to increase the number of IPs within the Scottish community setting [25] and from 2026 all pharmacists in the UK will be trained as IPs by the time they join the GPhC register. To meet these challenges an increased number of DPPs will be required and innovative solutions to this will need to be considered.

An issue related to the CFIR construct ‘characteristic of individuals’ in this work was that there may be a lack of *‘*self-efficacy’ around abilities to effectively negotiate time, role backfill, and other operational resources in order to undertake the role effectively. This has been previously identified as a concern in relation to the development of NMP practice [45]. A further ‘self-efficacy’ related matter focussed on collaborative practice which is essential for effective modern healthcare practice in all settings [40]. Interprofessional working has been shown to be important for community pharmacists in relation to medication safety and prescribing practice [46].

In 2020, the Scottish Government and CP Scotland allocated funding to support pharmacy contractors with the NHS Pharmacy First Plus service. Money was also set aside towards funding educational infrastructure to increase the number of independent prescribers within the Scottish community setting [23, 25].

## Recommedations for further research

Future work needs to explore the views of relevant stakeholders, especially other healthcare professions, such as medical doctors and nurses, who could act in the DPP role. Further exploration of the barriers and facilitators for implementation of the DPP role is also required. Lastly, there is an educational need to further define and scope the education and training required for community pharmacist prescribers taking on the DPP role.

## Conclusion

Overall, questionnaire respondents positively viewed the DPP role and supported its development. This research has provided original findings which can support implementation of the RPS DPP Framework. Further research is required to explore the implementation and consolidation of this model of preceptorship into normal models of practice.
